# The association between the adenoid microbiome and chronic otitis media with effusion in children differs according to age

**DOI:** 10.3389/fcimb.2025.1660939

**Published:** 2025-10-17

**Authors:** Jae-Won Jo, Sung Kyun Kim, Jae Yong Byun, Seok Min Hong, Bong-Soo Kim

**Affiliations:** ^1^ Department of Life Science, Multidisciplinary Genome Institute, Hallym University, Chuncheon, Gangwon-do, Republic of Korea; ^2^ Department of Nutritional Science and Food Management, Ewha Womans University, Seoul, Republic of Korea; ^3^ Department of Otolaryngology-Head and Neck Surgery, Kresge Hearing Research Institute, University of Michigan Medical School, Ann Arbor, MI, United States; ^4^ Department of Otolaryngology-Head and Neck Surgery, Kyung Hee University Hospital at Gangdong, Seoul, Republic of Korea

**Keywords:** adenoid, microbiome, chronic otitis media with effusion, children, age

## Abstract

**Introduction:**

Chronic otitis media with effusion (COME) can adversely affect childhood development, and while the adenoid has been considered a reservoir for bacterial pathogens contributing to the pathogenesis of COME, the role of the adenoid microbiome in COME remains unclear. This study analyzed both the adenoid and gut microbiome in children with and without COME to identify their potential roles in the disease’s pathogenesis.

**Methods:**

Adenoid samples were collected during surgery for adenoid microbiome analysis, while fecal samples were collected for gut microbiome analysis. Microbiome was analyzed using whole metagenome sequencing and subsequent bioinformatic analysis.

**Results:**

A significant association between the adenoid microbiome and COME was detected, while no such association observed for the gut microbiome. The adenoid microbiome varied by age in the control group, but this age-dependent variation was perturbed in the COME group. Notably, in children aged 6–12 years, the adenoid microbiome was significantly associated with COME based on the type of middle ear fluid, where *Streptococcus pneumoniae* and *Haemophilus influenzae* were prominent indicators in the mucoid form of COME. The proliferation of these species in mucoid COME group was correlated with indicators for the serous COME group. The altered microbiome in COME patients may influence immune responses through the synthesis of spermidine and acetate, contributing to disease development.

**Discussion:**

This study highlights the age-dependent contribution of the adenoid microbiome–particularly in children aged 6 to 12 years–to the pathogenesis of COME.

## 1 Introduction

Otitis media with effusion (OME) is a common childhood disease defined as the presence of fluid in the middle ear without symptoms or signs of acute inflammation (Rosenfeld et al., 2016). Although most episodes of OME resolve quickly without complications, chronic OME (lasting more than 3 months; COME) can lead to significant developmental consequences, including hearing loss, language delay, and balance disorders (Monasta et al., 2012). Additionally, the financial burden on families and healthcare providers is substantial (Rosenfeld et al., 2016; Monasta et al., 2012). The pathogenesis of OME is multifactorial, involving anatomical, immunological, genetic, microbial, and environmental factors (Rovers et al., 2004; Marseglia et al., 2008; Saylam et al., 2010). Among these, the dysfunction of the eustachian tube (ET) is recognized as a central mechanism, which leads to abnormal ear fluid drainage and inflammation due to bacterial infection (Minovi and Dazert, 2014; Miura et al., 2012).

The adenoids, located at the nasopharyngeal entrance of the ET, are a critical reservoir of microbes and immune cells (Chan et al., 2017; Ari et al., 2019; Sokolovs-Karijs et al., 2024). The ‘adenoidal reservoir’ hypothesis proposes that resident microbes on the adenoid mucosal surface–particularly otopathogens such as *Streptococcus pneumoniae*, *Haemophilus influenzae*, and *Moraxella catarrhalis*–contribute to the persistence of OME (Johnston et al., 2019; Jacquier et al., 2020; Kim et al., 2019). Adenoidectomy has been shown to benefit children with OME regardless of adenoid size, further supporting a pathogenic role of adenoid microbiota (Wang et al., 2014; Kania et al., 2019). Importantly, adenoidal microbiota are not static; they undergo age-dependent changes associated with adenoid hypertrophy and the maturation of ET function. These developmental processes are thought to influence both the composition of adenoidal microbial communities and their role in OME persistence (Papaioannou et al., 2013). However, few studies have explicitly investigated the interaction between age, adenoidal microbiota, and COME.

Beyond adenoidal colonization, the middle ear effusion (MEF) itself exhibits heterogeneity. Effusions are generally classified as serous or mucoid, with mucoid effusions characterized by higher viscosity and enriched in immune mediator, often linked to worse outcomes (Hurst and Venge, 2002; Dodson et al., 2012). The biological basis of these effusion subtypes, and their relationship to adenoidal microbiota, remains poorly defined.

Previous studies have mainly relied on culture-based methods or 16S rRNA amplicon sequencing, which provided relatively limited taxonomic resolution and functional information. Whole metagenome sequencing (WMS) offers an opportunity to characterize microbiome at higher resolution, enabling simultaneous analysis of taxonomic composition, functional pathways, and microbial interactions (Bogaert et al., 2011; Franzosa et al., 2015). This is particularly relevant for understanding whether altered microbial metabolism–such as shifts in immune-modulating metabolites–contributes to COME.

The mucosal immune system also plays a significant role in the inflammatory response observed in patients with OME. Although the immune cells in the middle ear and adenoid directly contribute to the abnormal immune response (Dodson et al., 2012; Brambilla et al., 2014; Wang et al., 2012), the gut microbiome also plays a critical role in the systemic immune response (Miyauchi et al., 2023; Clemente et al., 2018). Several studies have explored the impact of the gut microbiome on respiratory diseases, leading to the proposal of the ‘gut-lung axis’ hypothesis (Durack et al., 2018; Fujimura et al., 2016; Dang and Marsland, 2019). However, whether such systemic interactions contribute to COME remain unclear.

In this study, we aimed to address these gaps by performing WMS of adenoidal tissue and stool samples from children with and without COME. We analyzed (i) age-dependent variation in the adenoidal microbiome, (ii) differences between mucoid and serous effusion subtypes, (iii) functional microbial features including the Kyoto Encyclopedia of Genes and Genomes (KEGG) Ortholog (KO) profile, and (iv) potential links between the gut microbiome and COME. By integrating these parameters, our study provides a comprehensive analysis of the local and systemic microbiome in COME pathogenesis.

## 2 Materials and methods

### 2.1 Ethical approval

Ethical approval was obtained from the Institutional Review Board (IRB) of Hallym University Dongtan Sacred Heart Hospital (IRB no. 2019-05-007-002). All steps of the study were conducted in accordance with the principle outlined in the Declaration of Helsinki concerning biomedical studies involving human participants. Written informed consent was obtained from the parents of each child.

### 2.2 Study design and inclusion criteria

The study was carried out as a prospective study between May 2020 and February 2021. The OME group consisted of children who solely required ventilation tube insertion. The inclusion criteria for ventilation tube insertion were the presence of COME persisting for > 3 months from the onset or diagnosis date. The control group consisted of children receiving tonsillectomy and/or adenoidectomy due to obstructive symptoms such as snoring and mouth breathing. Patients who had ingested oral antibiotics within 4 weeks before sample collection were excluded from the study.

### 2.3 Sample collection

Adenoid samples were collected during surgery under general anesthesia at Hallym University Dongtan Sacred Heart Hospital. To prevent contamination of the adenoid samples, we observed the adenoid with direct vision using a 70-degree rigid telescope (KARL STORZ, Tuttlingen, Germany) when the ventilation tube insertion was performed. The adenoid size was evaluated using an endoscopic grading scale as per the study by Cassano et al. that delineated adenoid enlargement into four distinct categories: grade 1, representing adenoid occupying less than 25% of the choanal area; grade 2, where the adenoids occupied 25% to 50% of the choanal area; grade 3, indicating adenoids occupying 50% to 75% of the choanal area; and grade 4, denoting adenoids occupying 75% to 100% of the choanal area (Cassano et al., 2003). Swabs were collected through an intraoral approach using sterile cotton (482CE; COPAN, Brescia, Italy). Collected swabs were placed in a collection tube and the remainder of the stem was discarded. The tubes were then capped and immediately stored at -80 °C until DNA extraction. Fecal samples were collected using an OMNIgene gut tube (DNA Genotek, Ontario, Canada) according to the manufacturer’s instruction. Collected fecal samples were immediately stored at -80 °C until DNA extraction. We examined clinical data related to COME, including a history of previous ventilation tube surgery, allergy testing (multiple allergens simultaneous test; MAST), the phenotype of middle ear effusion (serous or mucoid), and the interval between the last antibiotic usage and sampling. If multiple class 1 adenoids tested positive for at least one antigen in the MAST, it was considered positive. The criteria for defining mucoid effusion include the presence of mucus strands upon gross examination and the absence of gravitational flow of the effusion (Val et al., 2018; Lin et al., 2003).

### 2.4 DNA extraction and WMS

Metagenomic DNA was extracted from adenoid tissue and fecal samples using the RNeasy PowerMicrobiome Kit (Qiagen, Inc., Valencia, CA, USA). The extracted DNA was purified using the DNeasy PowerClean Pro Cleanup Kit (Qiagen) according to the manufacturer’s instructions. The DNA concentration was measured using the BioPhotometer D30 with a μCuvette G1.0 (Eppendorf, Hamburg, Germany). To eliminate potential contaminations during the experimental process of sequencing-based studies (Eisenhofer et al., 2019), negative controls were included at every step. A total of 15 negative samples were sequenced along with the samples. These comprised sampling tubes, DNA-free water added to the DNA extraction kit, DNA-free water added to the purification kit, and DNA-free water added to the sequencing library preparation kit.

The DNA extracted from adenoidal (n=33) and fecal samples (n=32) were used to prepare the WMS library using a Swift 2S™ Turbo DNA Library Kit with Adapter (Swift Biosciences, Ann Arbor, MI, USA) following the manufacturer’s instruction. Index polymerase chain reaction (PCR) was performed using a 2S Combinatorial Dual Indexing Kit (Swift Biosciences). The library size was verified using a Bioanalyzer 2100 (Agilent Technologies, Santa Clara, CA, USA). The concentration of the library was measured using Qubit™ dsDNA HS Assay Kits (Thermo Fisher Scientific, Waltham, MA, USA). The equimolar concentration of each library was calculated using a TaKaRa PCR Thermal Cycler Dice Real Time System III (TaKaRa Bio, Inc., Shiga, Japan) with the GenNext NGS Library Quantification Kit (Toyobo, Osaka, Japan). Libraries were pooled and sequenced using the Illumina NovaSeq system (250-bp paired ends).

Sequence reads obtained from the NovaSeq system were analyzed as described previously (Lee et al., 2018, 2022). Adapter removal and quality filtering were performed using Trimmomatic with default options (Bolger et al., 2014). Paired-end sequences were merged using PEAR v.0.9.11 (Zhang et al., 2014). Human gene features were removed using BBMap with a reference human genome (HG-19). Taxonomic profiles were obtained using MetaPhlAn v.3.0 (Segata et al., 2012), and functional profiles were obtained using HuMAnN v.3.0 (Beghini et al., 2021). The resultant UniRef90 IDs were converted to KO. A total of 610,003,360 reads were obtained from the WMS analysis.

### 2.5 Quantification of bacterial amounts in samples

Total bacterial amounts in collected samples were estimated using quantitative real-time PCR based on the 16S rRNA gene, as described previously (Lee et al., 2022). The DNA extracted from samples was amplified in a 25-μL reaction mixture containing 12.5 μL of TB Green Premix Ex Taq (Tli RNaseH Plus) (TaKaRa Bio, Inc.), 2 μM of each primer, and 1 μL of DNA template (a 10-fold dilution series of sample DNA) with the primer 340F (5′-TCC TAC GGG AGG CAG CAG-3′) and 518R (5′-ATT ACC GCG GCT GCT GG-3′) with a Thermal Cycle Dice Real-Time System III (TaKaRa Bio, Inc.). The amplification conditions were 95 °C for 30 s, followed by 40 cycles of denaturation at 95 °C for 5 s and annealing at 60 °C for 30 s. Each sample was measured in triplicates. We quantified the bacterial amount by comparing threshold cycle values to a standard curve generated from parallel reactions of serial dilutions (1 × 10^1^- 1 × 10^7^) of the 16S rRNA gene from the *Escherichia coli* K12 w3110 strain. Regression coefficients (r^2^) for all standard curves were ≥ 0.99.

### 2.6 Microbiome data analysis

To mitigate potential contamination during the experimental process, decontamination was performed using the R package ‘decontam’ based on the sequences of the negative controls (Davis et al., 2018). Decontamination was performed with a threshold of 0.5 prevalence in the ‘isContaminant’ function. After decontamination, the analyzed features were selected based on a > 10% sample prevalence to increase the relevance of the results.

The Shannon diversity index was calculated using the R package ‘microbiome’. The principal coordinates analysis (PCoA) plot was obtained based on Bray-Curtis dissimilarity.

The influence of covariates, including age, sex, date of last antibiotic administration, duration of COME, adenoid size, allergies, and tube surgery, on the differences in the microbiome was analyzed using the ‘envfit’ function in the R package ‘vegan’ (v.2.5-7). The significance of covariates was calculated using permutational multivariate analysis of variance (PERMANOVA), and *p-value* < 0.05 were considered statistically significant.

Multivariate regression analysis was performed using multivariate association with linear models (MaAsLin2) (Mallick et al., 2021). The taxonomic and functional features were normalized by relative abundance and cumulative sum scaling (CSS) methods, respectively.

The important species for determining the microbiota age were selected through random forest regression with the ‘randomForest’ R package. The chronological age of the microbiota in children without COME was used as a training set to estimate the microbiota age (Galazzo et al., 2020). The N top discriminatory species were identified using the ‘rfcv’ function in the randomForest package. The increased node impurity index (IncNodePurity) to determine the importance of discriminatory species was calculated using the ‘importance’ function in the randomForest package. The relative abundance of important species was compared among groups and visualized in a heatmap using the ‘ggplot2’ R package. Spearman’s correlation was used to analyze the correlation between the estimated microbiota age and chronological age.

The indicator species for each group (control, serous-, and mucoid-COME) in children aged 6–12 years were determined using the ‘multipatt’ function of the ‘indicspecies’ R package with 10,000 permutations. Results with *p* < 0.05 and an indicator value > 0.5 were considered indicators for each group. The indicator species are presumed to be the main contributors to the observed microbiota differences across the groups.

The interspecies correlations in adenoid microbiota were analyzed using FastSpar with 1,000 bootstraps (Watts et al., 2019). The corresponding correlation network was visualized using the ‘qgraph’ R package. Significant correlations with p < 0.05 and a correlation value < |0.2| were demonstrated in the network.

Functional features in the adenoid microbiome were analyzed based on the KO category. The relative abundance values, represented as Reads Per Kilobase (RPK), for the KO categories and gene families, were normalized using the CSS method. The KO categories and gene families were compared between groups using MaAsLin2. Functional features between groups were depicted visually on volcano plots to highlight significant differences. The x-axis displays the fold-change of gene families (log_2_ fold-change), and the y-axis demonstrates significance with -log_10_ (*p-value*) calculated using MaAsLin2. Pearson’s correlation analysis was conducted to explore the relationship between indicators for mucoid COME and significantly different gene families (*p* < 0.001).

### 2.7 Statistical analysis

The variables of clinical data between groups were compared using a two-sided Fisher’s exact test. The differences in taxonomic and functional features between groups were determined using the Wilcoxon-rank sum test in the R software. Dunn’s multiple comparison tests were used to identify the differences among groups using the ‘dunn.test’ R package. The Benjamini-Hochberg false discovery rate multiple testing correction was utilized to adjust the *p-value* where applicable. The beta-diversity of taxonomic and functional features was visualized using the PCoA plots based on Bray-Curtis dissimilarity, and significance was determined using PERMANOVA (Adonis from the package vegan with 999 permutations). Results with *p* < 0.05 and *q* (adjusted *p-value*) < 0.05 were considered statistically significant.

## 3 Results

### 3.1 Sample characteristics and covariate analyses revealed significant determinants of adenoidal microbiota variation

The number of participants, age, and sex were not significantly different between the control and COME patient groups (**Table 1**). We analyzed 80 WMS data (adenoidal sample = 33, fecal sample = 32, and negative control sample = 15) in this study. The Decontam pipeline was utilized to trim potential contaminant sequences, identified based on the sequences detected in 15 negative controls, encompassing the sampling to library preparation processes (**Supplementary Figure 1**). Microbiota detected in negative samples significantly differed from those in adenoidal and fecal samples (*p* < 0.001). Further microbiome analyses were performed after removing potential contaminants.

**Table 1:** Clinical characteristics of the study participants.

Phenotype	Adenoid sample (n= 33)			Fecal sample (n= 32)		
	Control	COME	*p-value*	Control	COME	*p-value*
Participants (n)	18	15	0.728	18	14	0.597
Age (years)	6.33 ± 2.43	6.13 ± 1.96	0.796	5.5 ± 1.42	6.43 ± 1.80	0.216
Sex (male/female)	7/11	7/8	0.732	5/13	6/8	0.465
History of previous ventilation tube surgery (yes/no)	–	5/10		–	5/9	
Adenoid size (+1/+2/+3/+4)	–	5/6/4/0		–	5/6/3/0	
MAST (+/-/unknown)	–	6/6/3		–	6/4/4	
Middle ear fluid						
Serous	–	7 (46.7%)		–	7 (50.0%)	
Mucoid	–	8 (53.3%)		–	7 (50.0%)	
Date from the last antibiotic usage before sampling						
30–40 day	–	9 (60.0%)		–	9 (64.3%)	
45–90 day	–	6 (40.0%)		–	5 (35.7%)	

COME, Chronic otitis media with effusion; –, Not determined; MAST, Multiple antigen simultaneous test.

The covariates significantly associated with the variation of microbiota were determined using the EnvFit model (**Supplementary Table 1**). The variation in adenoid microbiota was observed to be associated with COME disease (r^2^ = 0.097, *p* < 0.05). Age was a significant factor influencing the composition of the adenoid microbiota in the control group (r^2^ = 0.415, *p* < 0.05). Conversely, in the COME group, the type of middle ear fluid (MEF), whether serous or mucoid, was identified to be a significant determinant of adenoid microbiota variation (r^2^ = 0.278, *p* < 0.05).

### 3.2 Adenoid and gut microbiota composition and diversity differed by COME status and MEF type

**Figure 1:**
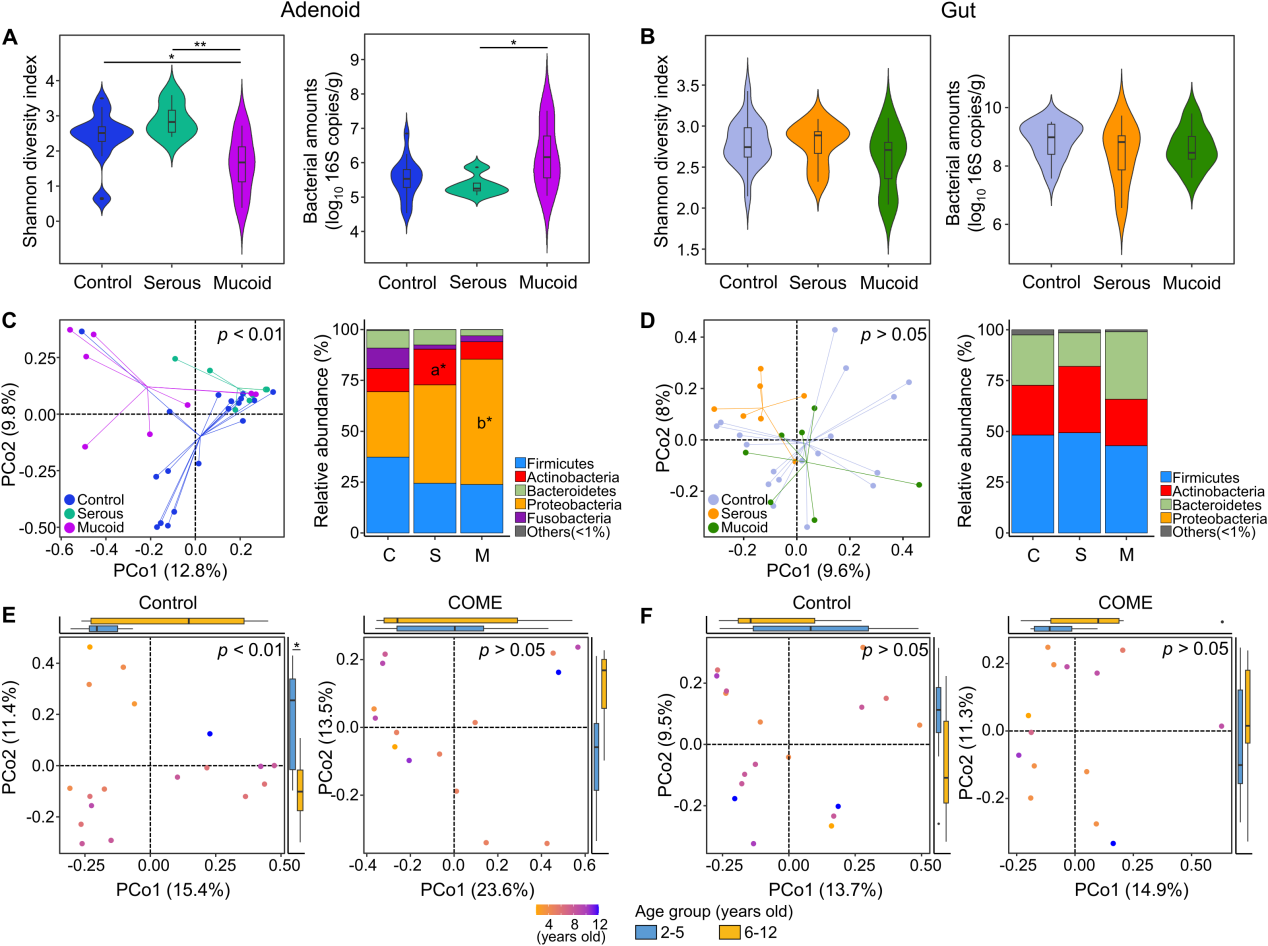
Comparison of adenoid and gut microbiota according to middle ear fluid (MEF) types. Bacterial diversity and amounts are compared according to MEF types in the **(A)** adenoid and **(B)** gut microbiome. The composition of microbiota in the **(C)** adenoid and **(D)** gut is compared between the control and COME groups in the PCoA plots based on the species-level Bray-Curtis dissimilarity and the bar plots at the phylum level. Age-dependent variations in **(E)** adenoid and **(F)** gut microbiota are compared in the control and COME groups. *p<0.05, **p<0.01, a*p<0.05 between control and serous groups, b*p<0.05 between control and mucoid groups. COME, Chronic otitis media with effusion; PCoA, Principal coordinates analysis.

The diversity of adenoid microbiota in COME with mucoid MEF was lower than those in the control and COME with serous MEF groups (*p* < 0.05; **Figure 1A**). On the other hand, the bacterial amounts in the adenoid microbiota of COME with mucoid MEF were higher than those in the other groups. The bacterial diversity and amounts in the gut microbiota did not differ significantly among the groups (**Figure 1B**). The adenoid microbiota demonstrated significant differences among the groups based on Bray-Curtis dissimilarity in the PCoA plot (*p* < 0.01; **Figure 1C**). This result was consistent with the influence of covariates as determined by the EnvFit model (**Supplementary Table 1**). Notably, Actinobacteria and Proteobacteria were identified as significantly different phyla between the groups. However, no significant differences were observed in gut microbiota among the groups in the PCoA plot or in terms of phylum composition (**Figure 1D**).

### 3.3 Age-dependent alterations in adenoid microbiota in relation to COME

Previous studies have reported the peak prevalence of OME between the ages of 2 and 5 years, coinciding with the period of rapid adenoid growth observed between the ages of 3 and 5 years (Dhooge et al., 2005; Papaioannou et al., 2013). Therefore, we analyzed the variation in microbiota according to age and compared the differences between the 2–5 years and 6–12 years age groups. In agreement with these previous studies, age was identified as a significant covariate in the adenoid microbiota of the control group. However, this age-dependent variation was not observed in the gut microbiota, but only in the adenoid microbiota of the control group (**Figures 1E, F**).

**Figure 2:**
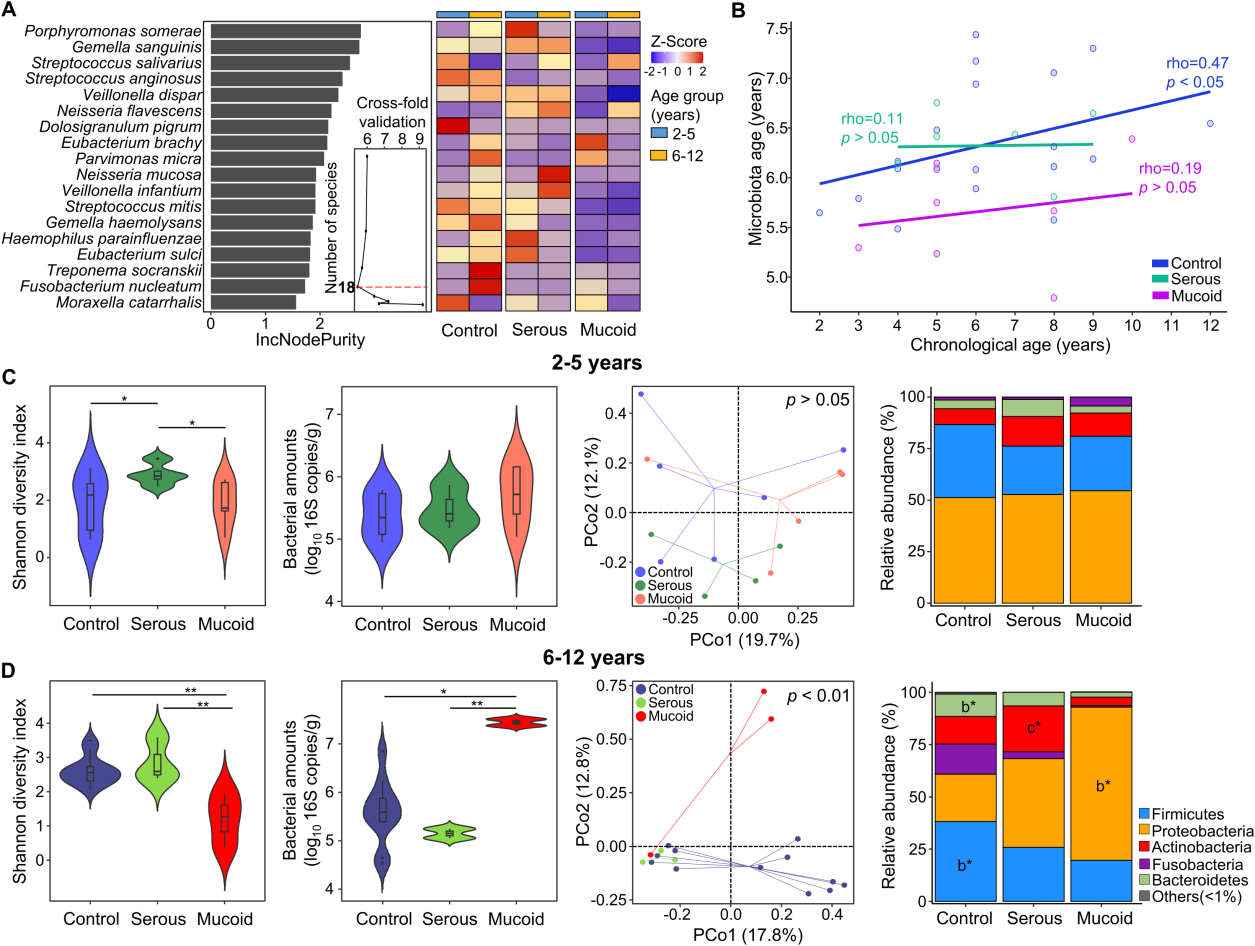
Perturbation of age-dependent variations in adenoid microbiota among COME groups. **(A)** Species importance in the prediction model of microbiota according to age within the control group. The 18 species with the highest discriminating power are selected by the lowest cross-validation error (inner graph). The heatmap displays the comparison of the relative abundance of 18 selected bacterial species across different age groups between the control and COME groups stratified by MEF types. **(B)** The correlation between chronological age and predicted adenoid microbiota age in the control and COME groups. **(C)** The microbiota and bacterial amounts are compared according to MEF types in children aged 2–5 years. **(D)** The microbiota and bacterial amounts are compared according to MEF types in children aged 6–12 years. *p<0.05, **p<0.01, b*p<0.05 between control and mucoid groups, c*p<0.05 between serous and mucoid groups. COME, Chronic otitis media with effusion; MEF, Middle ear fluid.

We analyzed the adenoid microbiota across different age groups. Random forest modeling after cross-fold validation identified eighteen species, including *Porphyromonas somerae* and *Gemella sanguinis*, as influential age-dependent species (**Figure 2A**). The relative abundances of these species according to age were perturbed in children with COME compared to those in controls. The predicted age of the adenoid microbiota was positively correlated with the chronological age in the control group (rho = 0.47, *p* < 0.05). In contrast, the adenoid microbiota in the COME group exhibited age-independent patterns (**Figure 2B**).

Our analysis revealed age-associated perturbations in the adenoid microbiota compositions among children with COME. The alteration in adenoid microbiota was analyzed according to the type of MEF within each age group. Although the diversity was significantly different between control and COME groups (*p* < 0.05), bacterial amounts and microbiota compositions did not differ among the groups according to MEF types in children aged 2–5 years (*p* > 0.05; **Figure 2C**). In contrast, alterations in microbiota corresponding to MEF types of COME were evident in children aged 6–12 years (**Figure 2D**). The diversity was lowest in the mucoid COME group compared to that in the other groups (*p* < 0.01), whereas the bacterial amounts were highest in the mucoid COME group (*p* < 0.05). The composition of microbiota exhibited significant differences according to MEF types in the PCoA plot (*p* < 0.01). The relative abundances of Bacteroidetes and Firmicutes were higher in the control group than in the mucoid COME group, whereas the relative abundance of Proteobacteria was higher in the mucoid COME group than in the control group (*p* < 0.05). Actinobacteria was higher in the serous COME group than in the mucoid COME group (*p* < 0.05). These results suggest that the adenoid microbiota in the COME group is differentially affected by the age of the children. In children aged 6–12 years, the composition of the adenoid microbiota differed significantly among COME groups depending on MEF type. The gut microbiota did not significantly differ among the groups in both age categories of 2–5- and 6–12-year-old children (**Supplementary Figure 2**).

### 3.4 Indicator species associated with MEF types in children aged 6–12 years

**Figure 3:**
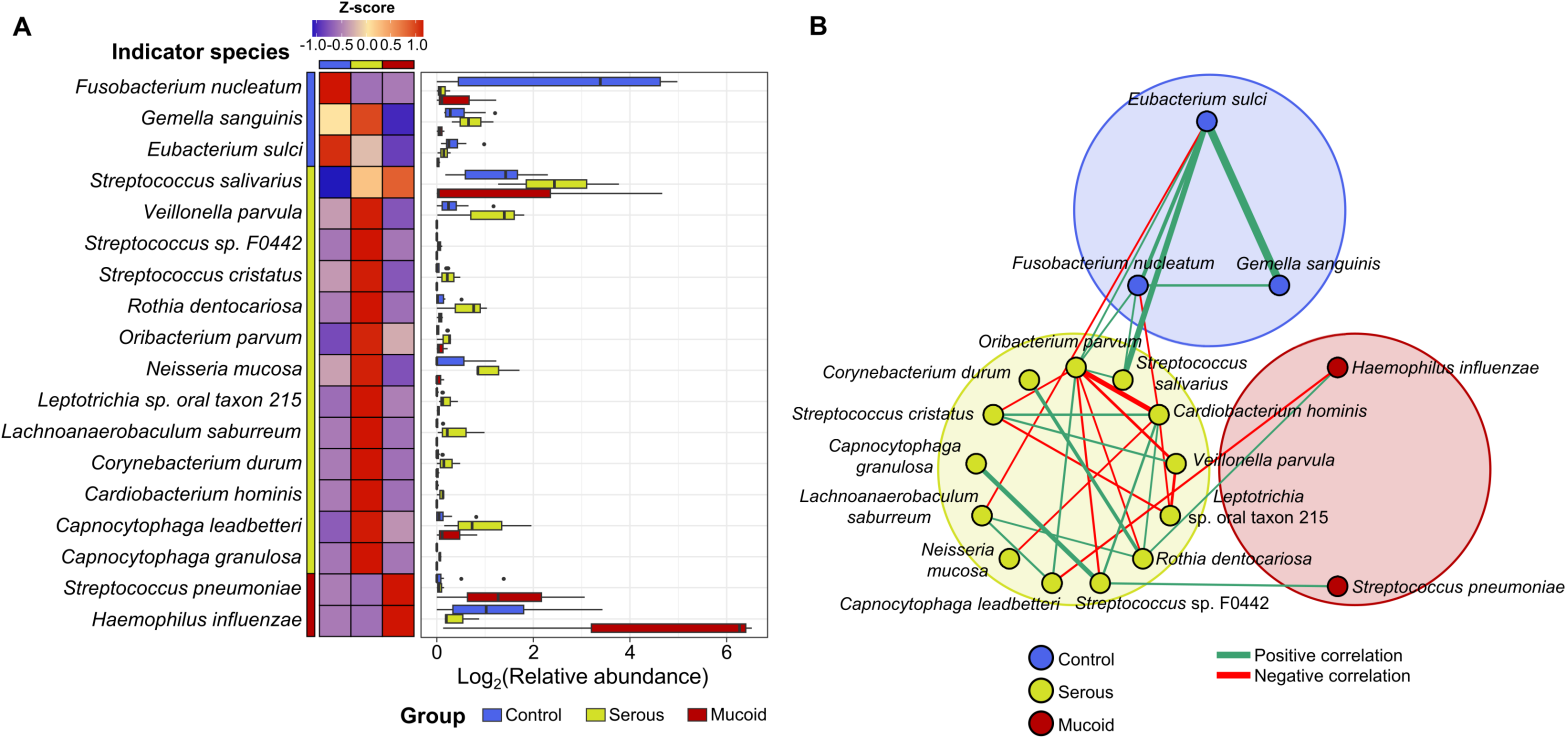
The difference in indicator species in the adenoid microbiota for each group. **(A)** The heatmap illustrates the comparison of the indicator’s relative abundance based on z-scores among groups. The relative abundances of indicators in each group are compared in the box plot. **(B)** The interactions between indicator species for each group are analyzed by the network analysis. Positive correlations are marked by green edges and negative correlations by red edges. Edge thickness denotes FastSpar correlation, ranging from -0.4 to 0.4. Only significant correlations with *p*<0.05 are demonstrated.

We analyzed the indicator species for each group in children aged 6–12 years to study the shift in adenoid microbiota according to MEF types. *Fusobacterium nucleatum*, *G. sanguinis*, and *Eubacterium sulci* were identified as indicators in the control group (**Figure 3A**). The relative abundances of *F. nucleatum* and *E. sulci* decreased in the COME group compared to those in the control group, whereas the relative abundance of *G. sanguinius* was higher in the serous COME group than in the other groups (*p* < 0.05). Thirteen species, including *Streptococcus salivarius*, *Veillonella parvula*, and *Neisseria mucosa*, were identified as indicators for the serous COME group, and their abundances were higher in the serous COME group than in the other groups (*p* < 0.05). *Streptococcus pneumoniae* and *H. influenzae* were indicators for the mucoid COME group. Although indicator species were commonly detected in all groups, their abundances varied across the groups.

These compositional differences in the relative abundances of indicator species across the groups could be partly driven by interactions among the indicators within each group. Significant positive correlations were observed among species within each group, except for those in the mucoid COME group (**Figure 3B**). Positive correlations were observed among the indicators of the control group, with higher correlation values than those noted in the other groups. Complex interactions between indicators were detected within the serous COME group, whereas indicators for the mucoid COME groups were not correlated with each other. Additionally, indicators for the serous COME groups were correlated with indicators for the control and mucoid COME groups. However, no direct correlation was identified between indicators for the control and mucoid COME groups. In the serous COME group, *N. mucosa* and *Cardiobacterium hominis* interacted negatively with *E. sulci* and *F. nucleatum* in the control group, respectively. This interaction could be associated with the reduced proportion of *E. sulci* and *F. nucleatum* in the serous COME group. *H. influenzae* in the mucoid COME group interacted positively with *Rothia dentocariosa* in the serous group, however, the organism interacted negatively with *Capnocytophaga leadbetteri*. *S. pneumoniae* in the mucoid COME group interacted positively with *Streptococcus* sp. F0442 in the serous COME group.

### 3.5 Functional features revealed potential influences of the altered adenoid microbiome in children aged 6–12 years with COME

**Figure 4:**
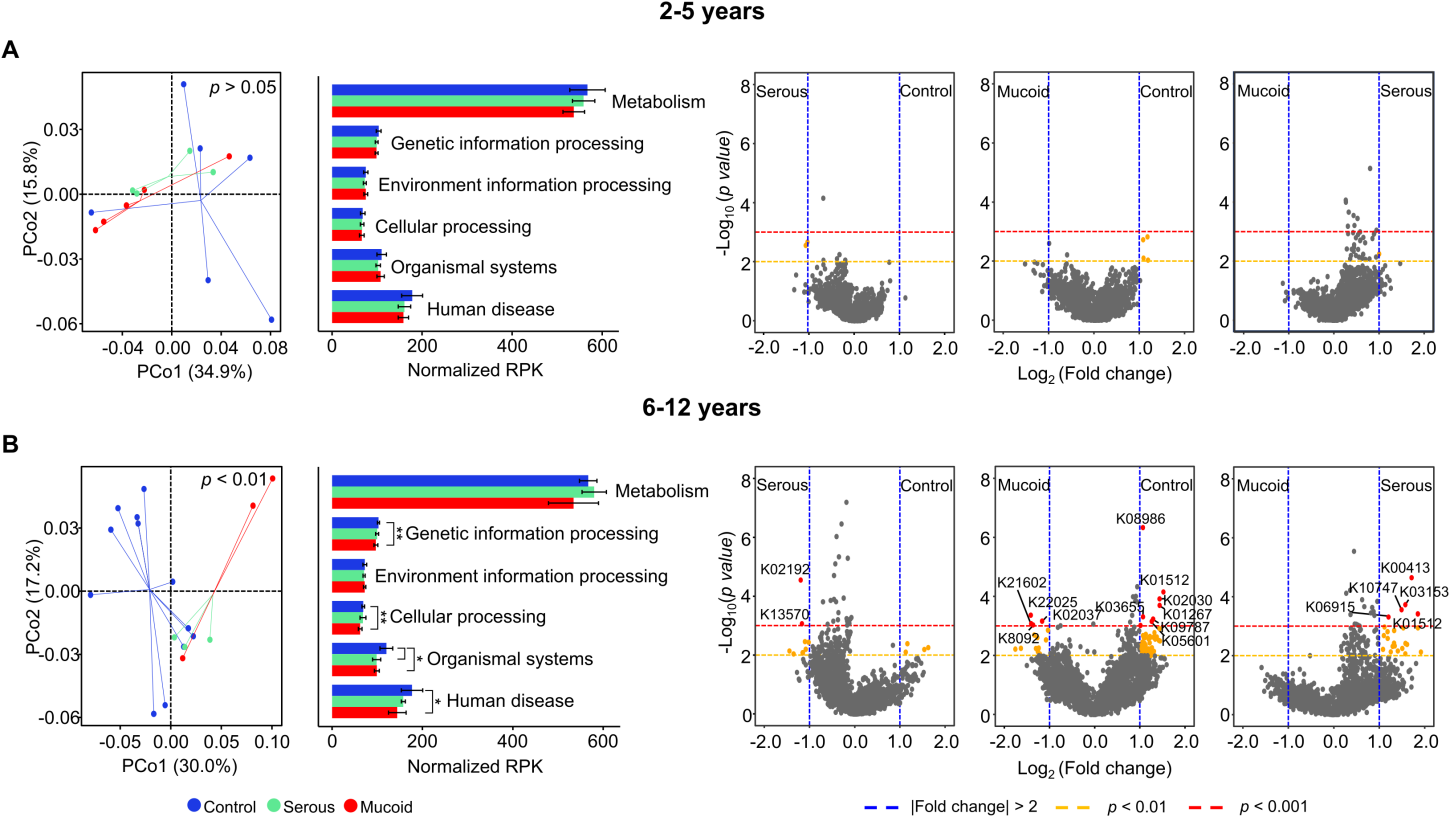
Comparison of functional features based on KEGG Orthology (KO) in the adenoid microbiome according to MEF types. Functional features in the adenoid microbiome are compared among groups of children aged **(A)** 2–5 years and **(B)** 6–12 years. The volcano plot displays the significantly different functional features of the adenoid microbiome between groups. Functional features with fold changes > |2.0| (blue dashed line), *p*<0.01 (yellow dashed line), and *p*<0.001 (red dashed line) are considered significant features. Only K numbers (gene family) with *p*<0.001 (red circle) are displayed. *p<0.05, **p<0.01. KEGG, Kyoto Encyclopedia of Genes and Genomes; MEF, Middle ear fluid.

The adenoid microbiome may play different roles in children with COME depending on the age group, as the microbiome demonstrates distinct alterations. To analyze the potential influence of the altered microbiome, functional features based on the KO were compared among groups within each age category. The functional features of the adenoid microbiome were not significantly different among the groups according to MEF types in the children aged 2–5 years (*p* > 0.05; **Figure 4A**). Although seven gene families differed among the groups (*p* < 0.01), they did not significantly contribute to the functional differences of the adenoid microbiome according to MEF types. However, the functional features of the adenoid microbiome significantly differed among groups within 6–12-year-old age group (*p* < 0.01; **Figure 4B**). A total of 120 gene families exhibited significant differences across the groups (fold changes > 2 and *p* < 0.01). Among these 120 significant features, 18 gene families had a *p-value* < 0.001. The number of features that differed between the control and mucoid COME groups (11 gene families with fold changes > 2 and *p* < 0.001) was higher than in the other comparisons (two gene families between control and serous COME groups; five gene families between serous and mucoid COME groups). These differences in functional features among the groups were consistent with previous microbiota results (**Figure 2**).

**Figure 5:**
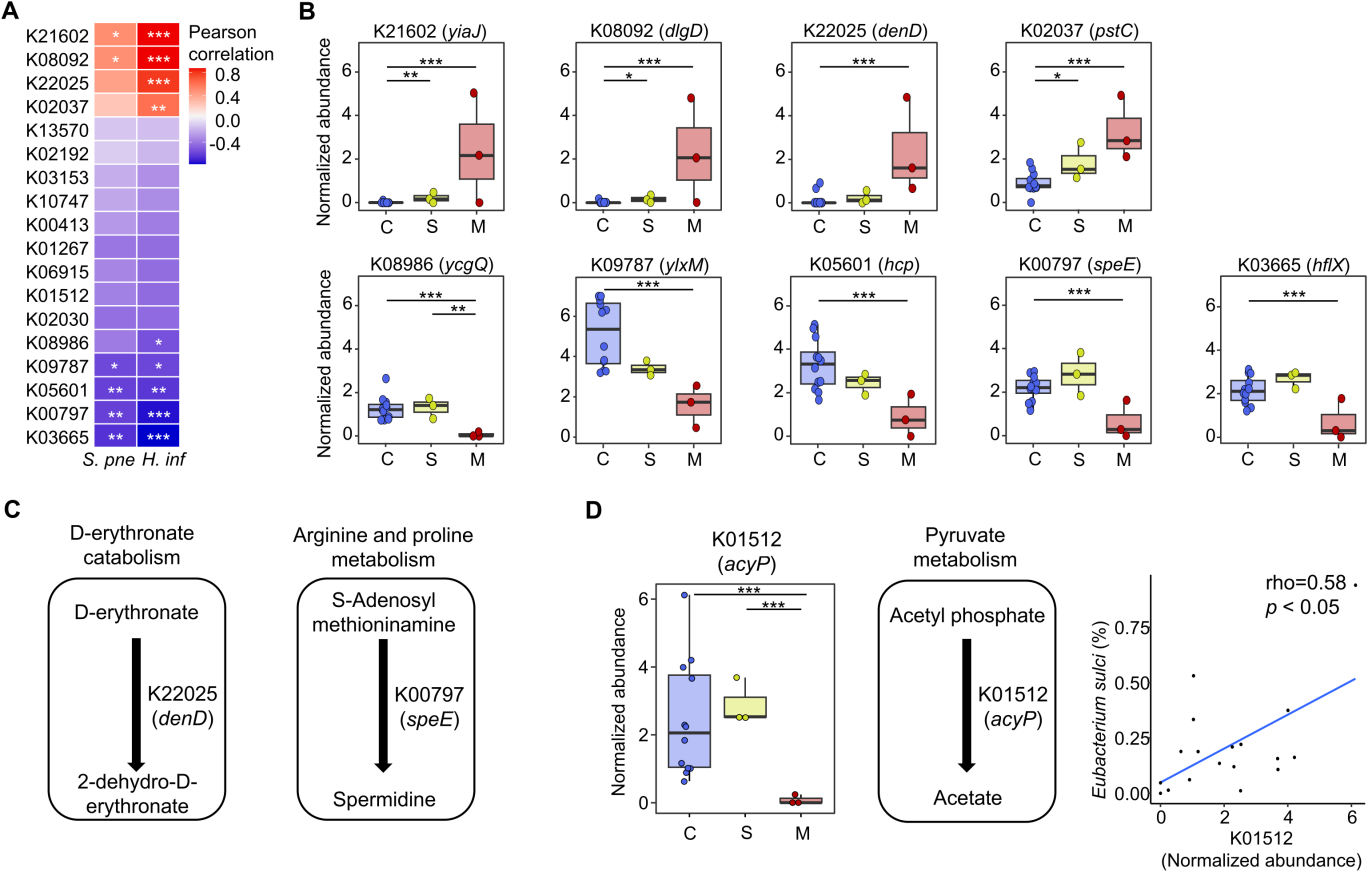
The correlated gene families with indicators for the mucoid COME group are compared among groups. **(A)** The correlation of significantly different gene families with *S. pneumoniae* and *H*. *influenzae* are analyzed by Pearson correlation analysis. **(B)** The normalized abundance of significantly correlated gene families with mucoid COME indicators is compared according to MEF types. **(C)** The reactions of significantly different gene families are demonstrated based on the KEGG database. **(D)** The commonly different gene families (K01512) in the mucoid COME group compared to those in the control and serous COME groups are detailed. The reactions are displayed based on the KEGG database. The correlation between the relative abundance of *Eubacterium sulci* and the normalized abundance of K01512 is significant (rho = 0.58, p<0.05). *p<0.05, **p<0.01, ***p<0.001. KEGG, Kyoto Encyclopedia of Genes and Genomes; *S. pne*, *S. pneumoniae*; *H*. *inf*, *H*. *influenzae*.

We analyzed the correlated gene families with indicators for the mucoid COME groups to identify potential functions of the altered adenoid microbiome. We identified nine gene families that were significantly correlated with *S. pneumoniae* and *H. influenzae* (*p* < 0.05; **Figure 5A**). Four gene families (K21602, K08092, K22025, and K02037) demonstrated a positive correlation with two indicators, and their abundances increased in the mucoid COME group (*p* < 0.05; **Figure 5B**). In contrast, five gene families (K08986, K09787, K05601, K00797, and K03665) exhibited a negative correlation with two indicators, and their abundances decreased in the mucoid COME group (*p* < 0.01). The D-erythronate catabolism pathway may be activated in the mucoid COME group through D-erythronate 2-dehydrogenase (*denD*; **Figure 5C**). The level of spermidine synthase (*speE*) was lower in the mucoid COME group compared to its level in the other groups.

In comparison to the control and serous COME groups, acylphosphatase (K01512; *acyP*) was identified to be a significantly different gene family in the adenoid microbiome of the mucoid COME group (*p* < 0.001; **Figure 4B**). The normalized abundance of *acyP* was lowest in the mucoid COME group (*p* < 0.001; **Figure 5D**), which may be related to the reduced production of acetate. This was associated with the reduced proportion of *E. sulci*, an indicator for the control group, in the adenoid microbiome of the mucoid COME group.

## 4 Discussion

We analyzed the microbiome in the adenoid tissue and gut samples obtained from children with and without COME to identify the influence of the microbiome in disease pathogenesis. To address limitations in previous studies, potential contaminations were removed from the analyzed sequence data, and whole metagenome sequences were used. The results revealed no significant difference in the gut microbiome between the control and COME groups across MEF types. In contrast, the association between the adenoid microbiome and COME exhibited age-dependent variations, with alterations in the adenoid microbiome displaying variability according to different MEF types.

An age-dependent variation in the adenoid microbiota was detected in control group, and these shifts were perturbed in children with COME. Several studies have reported microbiome changes with aging (Stewart et al., 2018; Aleman and Valenzano, 2019), and the development of the gut microbiome in early life is critical to host development, including the immune system (Al Nabhani and Eberl, 2020; Pronovost and Hsiae, 2019). Furthermore, host development can influence the development of the microbiome (Zhang et al., 2022; Lee et al., 2022). The adenoid microbiome consists of an ecosystem present on the mucosal surface of the adenoid. As the size of the adenoid decreases with age, the environment of the adenoid microbiome changes, which could be related to age-dependent variations in the adenoid microbiota (Papaioannou et al., 2013). Our study identified significant microbiome differences between MEF types in children aged 6–12 years, but not in those aged 2–5 years. This distinction may be partly explained by the maturation of ET function, which generally improves with age. Therefore, our findings suggest that in children aged 6–12 years, the adenoid microbiome may play an additional and more pronounced role in the pathogenesis of COME, beyond ET dysfunction alone. The lack of observed microbiota differences in previous studies comparing OME and control groups (Xu et al., 2020; Chan et al., 2016), can potentially be explained on the basis of not accounting for these age-dependent variations.

Middle ear effusions in COME are typically classified as either mucoid, characterized by high viscous and stickiness, or serous, which is less viscous. Mucoid effusions are largely composed of innate immune mediators, many of which are derived from neutrophils (Hurst and Venge, 2002). Although the underlying biological mechanisms driving the differences between these two effusion types remain poorly understood, clinical studies have reported that serous effusions are generally associated with more favorable treatment outcomes compared to mucoid effusions (Dodson et al., 2012). Furthermore, evidence suggests that as the duration of otitis media increases, mucoid effusions become more prevalent, supporting the hypothesis that effusions may transition from a serous to a mucoid state as the disease progresses (Matkovic et al., 2007).

In children aged 6–12 years, the diversity of adenoid microbiota was lowest in the mucoid COME group compared to that in the other groups, whereas the bacterial amounts were highest in the mucoid COME group. The relative abundances of *S. pneumoniae* and *H. influenzae* were higher in the mucoid COME group compared to those in the other groups and were identified as indicators for the mucoid COME group. These results indicate that *S. pneumoniae* and *H. influenzae* proliferate while other species are reduced in the adenoid of the mucoid COME group, resulting in a decreased diversity. These findings are consistent with the dominance of *Streptococcus* and *Haemophilus* in the adenoid of patients with OME (Chan et al., 2016; Xu et al., 2020). These two species interact with indicators of the serous COME group (*C. leadbetteri*, *R. dentocariosa*, and *Streptococcus* sp. F0042) in the species interaction network analysis. However, the indicators for the control group did not directly interact with the aforementioned two species. *Oribacterium parvum* displayed a positive correlation with the indicators for the control group and a negative correlation with *C. hominis*, which in turn correlated positively with *R. dentocariosa* and *Streptococcus* sp. F0042. These findings suggest that *O. parvum* may serve as a regulator species in microbiota transitions, though this interpretation requires caution given the cross-sectional design. The reduction of *O. parvum* may lead to an increase in *C. hominis*, with increasing *R. dentocariosa* and *Streptococcus* sp. F0042 in the serous COME group. This may be associated with the proliferation of *S. pneumoniae* and *H. influenzae* in the mucoid COME group. These findings suggest that the alteration of adenoid microbiota can be linked to the progression of COME.

Although further validation is necessary, our study detects distinct functional features of the adenoidal microbiome in children with the mucoid form of COME. The high abundance of *denD* (K22025) in the mucoid COME group may be associated with the activation of the D-erythronate catabolism pathway. D-erythronate is a metabolite of N-acetyl-D-glucosamine and is prevalent in human biofluids (Guo et al., 2024). Therefore, the high abundance of *denD* could be related to the MEF in the mucoid COME group. The decreased level of *speE* (K00797) may be related to the deficiency of spermidine in the mucoid COME group. Spermidine can be produced through the co-metabolism of host microbiota, and its deficiency is linked to various disorders, including immune dysfunction and inflammation (Madeo et al., 2018; Chamoto et al., 2023). Furthermore, the lowest level of *acyP* (K01512) may be associated with the insufficient level of acetate in the mucoid COME group. Acetate is an immune modulator that can affect the innate immune response to pathogenic bacteria such as *H. influenzae* and *S. pneumoniae* (Hosmer et al., 2022; MaChado et al., 2022). Therefore, the altered adenoid microbiome in the mucoid COME group can reduce the level of spermidine and acetate, thus enhancing inflammation and immune disorders associated with the proliferation of *H. influenzae* and *S. pneumoniae.*

The age-specific effects observed in our study may be linked to developmental changes in immune maturation and ET function. By stratifying analyses by age, our study reconciles discrepancies with previous findings (Chan et al., 2016; Xu et al., 2020). Clinically, microbial profiling of adenoidal tissue, particularly in older children, may hold potential as a biomarker to guide treatment strategies, though validation in larger longitudinal cohorts is essential.

In contrast to adenoid microbiome, no significant association between the gut microbiome and COME was found in present study. This suggests that local adenoidal rather than distal gut microbial influences play a predominant role in disease pathogenesis. However, further longitudinal studies are required to evaluate whether systemic gut-lung interactions contribute to COME under specific conditions.

The main limitations of this study include the relatively small number of participants in each group and the cross-sectional design, which precludes causal inference. In addition, we were unable to obtain detailed information on antibiotic exposure and allergen sensitization, which represent important potential confounders. This limitation underscores the need for longitudinal cohort studies with comprehensive clinical metadata to validate and expand upon our findings. However, it should be noted that the adenoid samples were collected during surgery under general anesthesia, which made it challenging to obtain a large number of samples. Nevertheless, this method was chosen to avoid contamination when collecting samples from the adenoids. Furthermore, the sample size analyzed in this study was comparable to those of previous studies (Xu et al., 2020; Chan et al., 2016; Sokolovs-Karijs et al., 2024).

In conclusion, the influence of the adenoid microbiome on COME is particularly relevant in children aged 6–12 years, a developmental period during which the adenoid size decreases and ET function improves compared to younger children aged 2–5 years. Alterations in the adenoid microbiome may interact with the host and contribute to the progression of COME. Specifically, *O. parvum* and *C. hominis* may play regulatory roles in the alteration of the adenoid microbiome in COME. The altered adenoid microbiome may be related to deficiencies in immune modulating metabolites such as spermidine and acetate. These findings suggest that alterations in the adenoid microbiome may be associated with COME in children aged 6–12 years, potentially indicating a distinct pathophysiology compared to otitis media in younger children under five years of age. Further studies are needed to elucidate the relationship between the potential functions identified in the adenoid microbiome and the pathogenesis of COME.

## Data Availability

The datasets presented in this study can be found in online repositories. The names of the repository/repositories and accession number(s) can be found below: https://www.ebi.ac.uk/ena, PRJEB72800.
