# Supplementary material for: The association between the adenoid microbiome and chronic otitis media with effusion in children differs according to age
Source: Front Cell Infect Microbiol. 2025 Oct 17;15:1660939. doi: 10.3389/fcimb.2025.1660939 (PMC12575324; doi:10.3389/fcimb.2025.1660939)
Supplement: Supplementary file 1 [file DataSheet1.pdf]

Supplementary Material

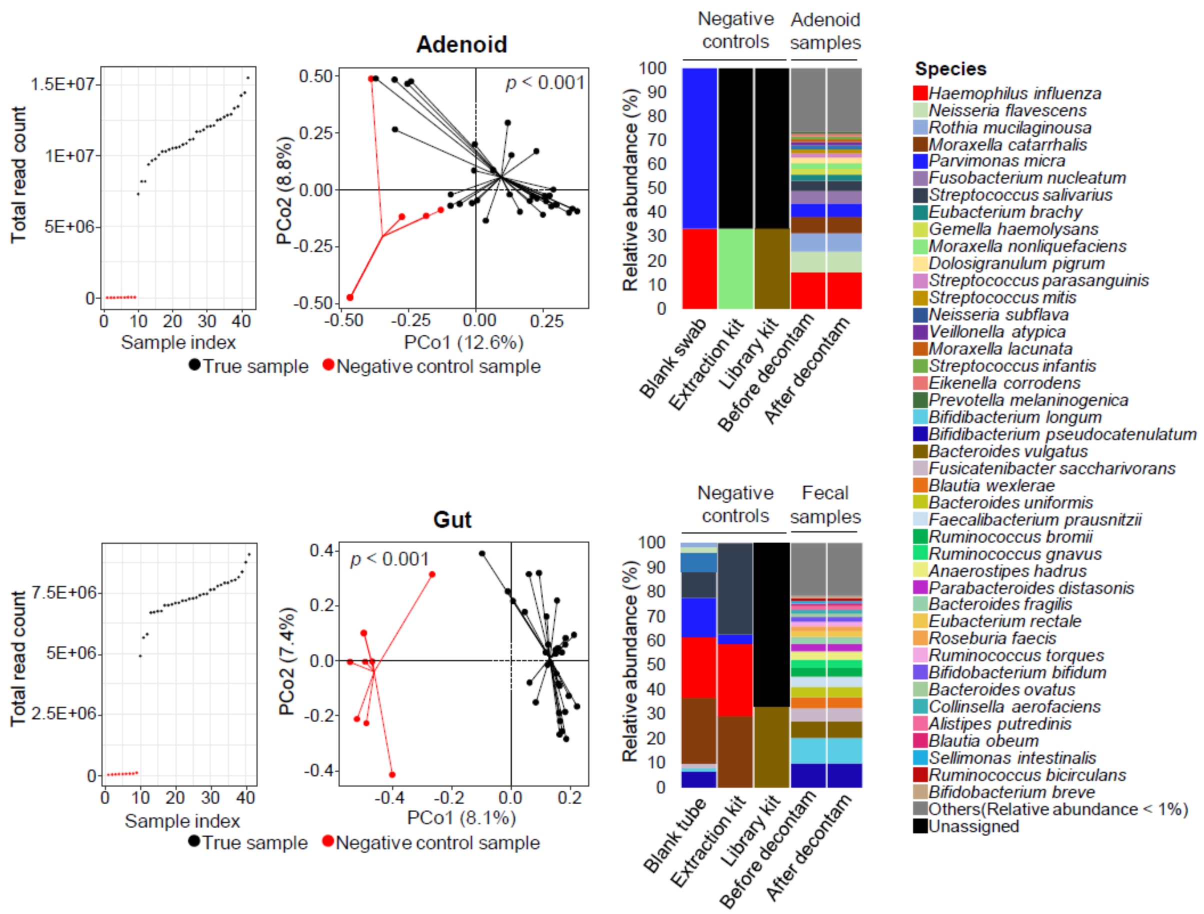

Supplementary Figure 1. Microbiota detected from sequences in negative controls. Negative controls included the sampling swab (blank), DNA-free water added to the DNA extraction kit, and DNA-free water added to the library preparation kit. The microbiota obtained from collected samples are compared with detected microbiota from negative controls based on Bray-Curtis dissimilarity in principal coordinated analysis (PCoA). Potential contaminants in the sequence data are removed based on detected sequences in negative controls by the Decontam pipeline..

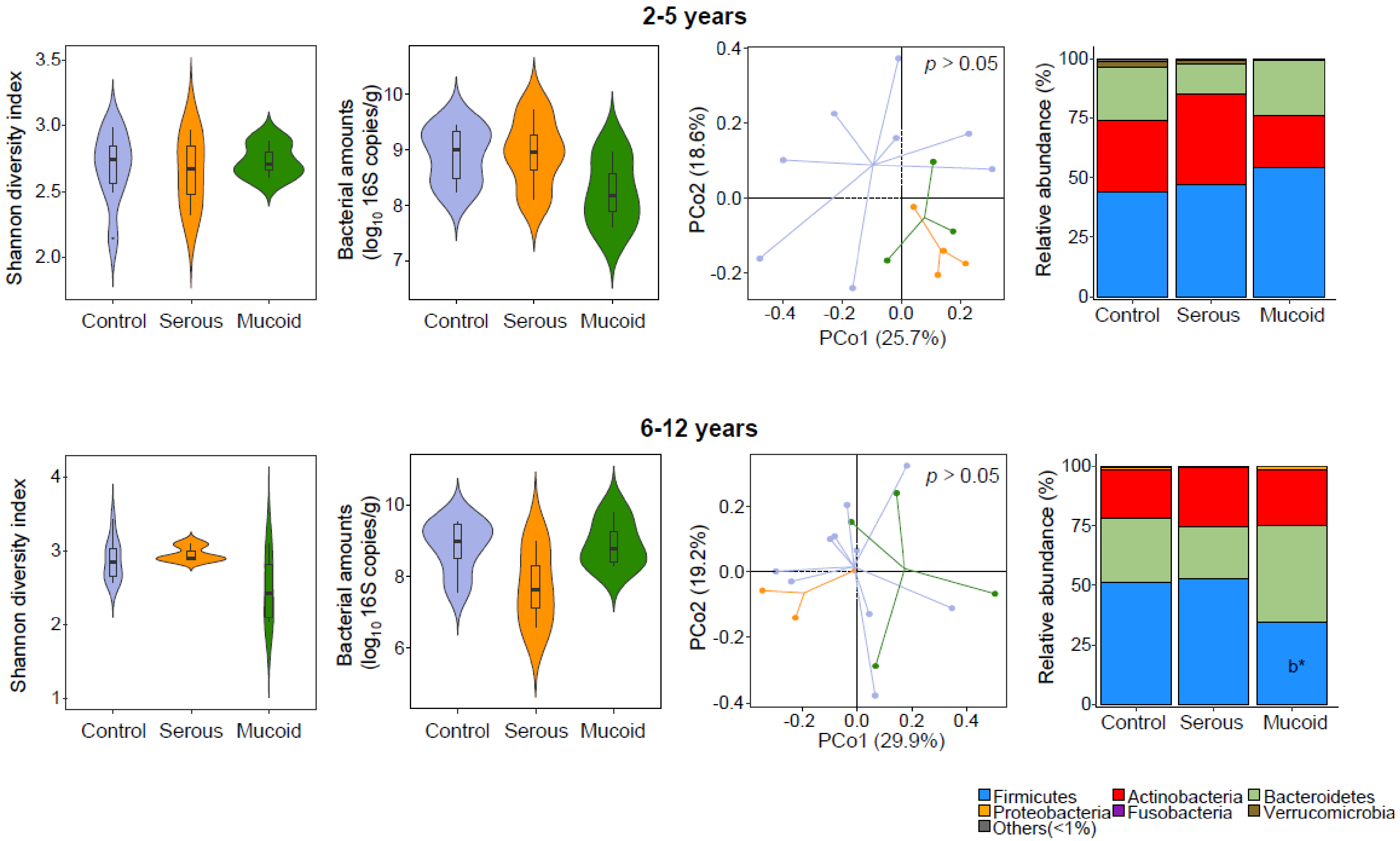

Supplementary Figure 2. Comparison of gut microbiota among groups according to middle ear fluid types in both 2–5- and 6–12-year-old children. b*$p < 0.05$ between control and mucoid groups.

Supplementary Table 1. Correlation between covariates and microbiota variation. The correlations were determined using the EnvFit model based on Bray-Curtis dissimilarity in each group.

		Adenoid sample		Fecal sample	
Group	Variable	R-squared	*p-value*	R-squared	*p-value*
Total	Age (years)	0.084	0.271	0.075	0.307
	Sex (male/female)	0.055	0.186	0.016	0.619
	Phenotype (control/COME)	0.097	0.037	0.025	0.481
	Middle ear fluid type (serous/mucoid)	0.243	0.003	0.132	0.077
Control	Age (years)	0.415	0.019	0.203	0.184
	Sex (male/female)	0.039	0.527	0.012	0.814
COME	Age (years)	0.201	0.261	0.137	0.451
	History of previous ventilation tube surgery (yes/no)	0.020	0.752	0.070	0.457
	Adenoid size (+1/+2/+3/+4)	0.092	0.553	0.055	0.737
	Middel ear fluid type (serous/mucoid)	0.278	0.025	0.252	0.017
	Date from the last antibiotic usage before sampling (30-40 day/45-90 day)	0.085	0.607	0.332	0.107

COME, Chronic otitis media with effusion.
